# ORY-1001 Delays Retinal Photoreceptor Degeneration in rd10 Mice by Inhibiting H3K4me2 Demethylation

**DOI:** 10.3390/biology15020132

**Published:** 2026-01-13

**Authors:** Xin Lu, Guang-Hua Peng

**Affiliations:** 1 Laboratory of Visual Cell Differentiation and Regulation, Basic Medical College, Zhengzhou University, Zhengzhou 450001, China; luxin@zzu.edu.cn; 2 Department of Pathophysiology, Basic Medical College, Zhengzhou University, Zhengzhou 450001, China

**Keywords:** ORY-1001, photoreceptor, rd10 mice, H3K4me2, demethylation

## Abstract

(1) Background: Modifications of histone methylation could alter chromatin structure and thereby have an impact on gene expressions. (2) Methods: To investigate whether ORY-1001 delay retinal photoreceptor degeneration, rd10 mice were intraperitoneally injected with ORY-1001 (0.075 mg/kg) every second day from the 14th to the 24th day after birth. Full-field electroretinogram detection (ff ERG), optical coherence tomography (OCT), visual behavioral testing, retinal tissue morphology observation, and protein expression detection experiments were performed on the 25th day. Simultaneously, ATAC-seq and RNA-seq were used to test the mice’s retinal tissues, and metabolomics detection and quantitative real-time polymerase chain reaction (qRT-PCR) were carried out. (3) Results: Compared with the rd10 group, in the treatment group, the function in the electroretinogram response and the visual behavioral responses were improved, the nuclear layer morphology of retinal tissue was reserved more, and the protein expression of H3K4me2 and CoREST was increased. Conjoint analysis of our ATAC-seq and RNA-seq results showed that chromatin accessibility was changed, as was gene expression which was involved in metabolism changes. In addition, the effector gene in the retina was Gnat1. (4) Conclusions: ORY-1001 delays retinal photoreceptor degeneration by inhibiting H3K4me2 demethylation in rd10 mice, which suggests that ORY-1001, as a novel epigenetic modifier, has potential for treating RP.

## Simple Summary

Our research shows that ORY-1001 delays the process of retinal photoreceptor degeneration in rd10 mice, a mouse model of retinitis pigmentosa (RP). To the best of our knowledge, besides treating acute myeloid leukemia and certain solid tumors in clinical trials, this is the first study to demonstrate that ORY-1001 is effective in treating RP in animal experiments, which is relevant for metabolism. Our primary results suggest that a new and novel epigenetic therapy for RP is possible, simply by inhibiting H3K4me2 demethylation.

## 1. Introduction

Retinitis pigmentosa (RP) is a disease that results in blindness due to the degeneration and sequential death of rod and cone cells, with a worldwide prevalence of 1/4000. It is associated with heavy disease burden and low life quality for patients and their families [1,2]. A typical RP symptom is night blindness, following concentric visual field loss, while rod photoreceptor dysfunction and central vision loss occurs later due to cone dysfunction. Electroretinograms show photoreceptor function reducing, and optical coherence tomography shows progressive loss of outer retinal layers [1,2]. However, due to genetic heterogeneity, caused by many gene mutations, effective treatments have been limited, and it is urgent to find new and effective treatments for RP patients. There are many similarities between the degeneration and death process of retinal photoreceptors in Pde6b^rd10^ (rd10) mouse that are caused by pde6b gene mutation and the pathogenesis and clinical symptoms of RP patients; therefore, as a mouse model of RP, the rd10 mouse provides possibility to evaluate potential therapies. The research progress in the field of epigenetics has provided new ideas and research methods for the treatment of retinitis pigmentosa. Histone methylation modification, a form of epigenetic regulation, can change chromatin accessibility and have a strong influence on gene expression by adding or removing methyl groups at specific sites on histones. It was found that demethylation of H3K4me2 is required for the transition from late progenitor to differentiated mouse rod photoreceptor, and H3K4me2 decreased during the process of degeneration and death of photoreceptors in rd10 mouse, but the cause of this is not clear yet [3,4]. The lysine-specific histone demethylase 1A (KDM1A) inhibitor ORY-1001, as a highly selective, irreversible, and specific histone demethylase inhibitor, show a strong ability to inhibit H3K4me2 from removing methyl and is safe and effective in treating acute leukemia and small cell lung cancer [5,6,7,8,9]. Therefore, we used ORY-1001 to inhibit the demethylation of H3K4me2 in order to study whether it can delay the degeneration of photoreceptors and to try to determine the changes in chromatin accessibility and gene expression.

## 2. Materials and Methods

### 2.1. Experimental Reagents

The KDM1A inhibitor Iadademstat dihydrochloride (ORY-1001 dihydrochloride) was purchased from Med Chem Express Company (Monmouth Junction, NJ, USA) (catalog 1431303-72-8). The main antibodies used were anti-rhodopsin (catalog ab98887, Abcam, Cambridge, UK), anti-KDM1A (catalog 2184S, CST, USA), anti-H3K4me2 (catalog 9725S, CST, USA), anti-HDAC1 (catalog 34589S, CST, Danvers, MA, USA), anti-HDAC2 (catalog 57156S, CST, Danvers, MA, USA), anti-H3 (catalog 4499S, CST, Danvers, MA, USA), anti-CoREST (catalog sc-376567, Santa Cruz, CA, USA), and anti-*β*-actin (catalog 66009-1-Ig, proteintech, Wuhan, China).

### 2.2. Experimental Instruments

The stereomicroscope (model: SZ61) and fluorescence microscope (model: BX53) were purchased from Olympus Corporation, Tokyo, Japan. The electrophysiological detection instruments for full-field electroretinogram recording (ff ERG) and optical coherence tomography (OCT) were purchased from Roland Consult Company, Falkensee, Germany. The visual behavioral recognition analysis equipment (Clever sys Top Scan) was purchased from Clever Sys Inc., Reston, VA, USA. The Q5 fluorescent quantitative PCR instrument was purchased from ABI Corporation, Santa Clara, CA, USA. The Western blotting analysis system, ChemiDoc Touch, was purchased from BioRad Company, Hercules, CA, USA. The frozen slicer Leica CM1950 was purchased from Leica Company, Wetzlar, Germany.

### 2.3. Experimental Animals and Treatment

The Pde6b^rd10^ (rd10) mice (RRID: MGI: 3581193) were purchased from Jackson Laboratory (Bar Harbor, ME, USA), while C57BL/6J Nifdc (wild-type, wt) mice were purchased from Vital River Laboratories (Beijing, China). All procedures (feeding, breeding, and treatments) were managed in strictly accordance with the Statement on the Use of Animals in Ophthalmic and Visual Research by the Association for Research in Vision and Ophthalmology and approved by the Animal Research Ethics Committee of Zhengzhou University (approval No. ZZUIRB2022-075) in order to minimize mice suffering. All mice were housed under standard conditions with a 12 h light/dark cycle, 40–60% air humidity, and 26 °C room temperature and provided with sufficient food (SPF-grade) and sterilized water. The mice were divided into three groups: wild-type (wt) (24 mice), rd10 (30 mice), and rd10+ORY (30 mice). The rd10 mice in the rd10+ORY group were injected intraperitoneally with ORY-1001 at a dose of 0.075 mg/kg every second day from P14 (postnatal) to P24, while the rd10 mice in the rd10 group and the wild-type mice in the wt group were injected intraperitoneally with isochoric 0.9% bacteriostatic sodium chloride instead. The drug ORY-1001 was diluted in saline.

### 2.4. Full-Field Electroretinogram Recording (ff ERG)

At P25, the mice were dark-adapted overnight before the ff ERG examinations. Under dim-red light conditions, they were placed on the examination table under isoflurane air anesthetizing, with pupil dilating, gold wire loop electrodes contacting their corneal surface, and steel needle electrodes being inserted into their skin and tails. Following this was stimulation with flashes of graded intensities of 3.0 cd/m^2^, and ERG data were recorded and collected via the amplifier of the Roland RETI-scan system. The a-wave amplitude was measured from baseline to the first trough, and b-wave amplitude was measured from the first trough to the next peak.

### 2.5. Optical Coherence Tomography (OCT)

At P25, the mice were anesthetized with isoflurane air, their pupils were dilated, and a specific convex lens was placed on their corneal surface to scan their retinal structures in vivo to record measurements with the Roland RETI-scan system OCT software RETI-port/scan 21. The images were collected and statistically analyzed at a distance of 200 μm from the optic nerve head.

### 2.6. Visual Behavioral Test: Dark–Light Transition Test

Two boxes of the same size were connected in the experimental device for the visual behavior dark–light transition test. The dark box was light–tight, the light box was placed under normal light, and there was a hole connecting the two boxes through which mice could enter and exit each box freely. At P25, the mice were placed in quiet and light environment for two hours. Then, they were placed one-by-one in the center of the light box and wandered at random. The analysis software recorded a mouse’s moving trajectory of movement in the light box and the percentage of the time that it spent in the dark box for a total of 6 min.

### 2.7. Immunofluorescence Staining

At P25, the mice’s eyeballs were quickly obtained and placed in a fixative fluid for 2 h and a 30% sucrose solution for 24 h. The anterior segment and lens nucleus were removed, and the rest was embedded with an embedding agent. Frozen sections were created using a freezing microtome (with a longitudinal thickness of 8 μm centered around the optic disk). The tissue on the section was washed with PBS solution and incubated with appropriate concentrations of primary (Rhodopsin) and secondary antibodies. The nuclei were labeled with DAPI and sealed. The images were collected and statistically analyzed under a fluorescence microscope at a distance of 200 μ m from the optic nerve head. The reagents used for immunofluorescence staining are shown in Table 1.

### 2.8. Western Blotting Analysis

Western blotting was performed by mixing the retinal tissue obtained at P25 with lysate to extract proteins, and then denaturing the proteins. Using electrophoresis, all the proteins were distributed and then transferred to a nitrocellulose membrane and incubated with the appropriate concentrations of primary and secondary antibodies, respectively (Table 2). Images were collected and statistically analyzed using the Western blotting analysis system ChemiDoc Touch (BioRad, Hercules, CA, USA).

### 2.9. ATAC-Seq (Chromatin with High-Throughput Sequencing)

Lysis buffer was added to the retinal tissue collected at P25 to extract cell nuclei, after which Tn5 transposase was added. The samples were incubated at 37 °C for 30 min, and then purified. PCR amplification was performed to obtain a DNA library, which was then sequenced using the sequencing platform Illumina HiSeq 2500. After quality inspection and annotation processing, the raw data were subjected to Kyoto Encyclopedia of Genes and Genomes (KEGG) analysis and Gene Ontology (GO) analysis to identify differentially expressed genes. Samples processing and data analysis were conducted by Wuhan SeqHealth Biotechnology Company, Wuhan, China.

### 2.10. RNA-Seq

Using magnetic beads, the mRNA was purified from RNA, which was extracted from retinal tissue collected at P25, and then amplified and purified by PCR. The samples were then sequenced on the Illumina HiSeq 2500 sequencing platform. After quality inspection and annotation processing, the raw data were subjected to KEGG analysis to identify differentially expressed genes. Sample processing and data analysis were conducted by Wuhan SeqHealth Biotechnology Company.

### 2.11. Metabolomics Detecting

At P25, retina tissue samples of the rd10 (short for Mrd) and rd10+ORY (short of Mrt) groups were collected for metabolomics detection using LS-MS (liquid chromatography–mass spectrometry) technology and analysis based on relevant databases. Under low-temperature conditions, homogenized beads were added, and an isotopically labeled extraction solution was added to methanol/acetonitrile/water = 2:2:1 (*v*/*v*) and vortexed for 30 s. The sample tubes were placed into the homogenizer, which was set at 35 Hz for 4 min to homogenize. Then, they were sonicated in an ice water bath for 5 min and the process was repeated 3 times. The samples were maintained at a low temperature for 1 h and then placed into a centrifuge set at 4 °C and 12,000 rpm for 15 min. Then the supernatant from the tubes was collected and transferred to new ones for detection. Target compounds were separated using Waters liquid chromatography columns and recorded as primary and secondary mass spectrometry data using an Orbitrap Exploris 120 mass spectrometer. All steps were performed by the Wuhan SeqHealth Biotechnology Company.

### 2.12. Quantitative Real-Time Polymerase Chain Reaction (qRT-PCR)

The extracted RNA was reverse transcribed to generate cDNA, and the selected target genes were amplified using the Q5 real-time fluorescence quantitative PCR instrument. The relative expression levels of the genes were calculated using the 2^−ΔΔCT^ method, and *β*-actin was used for normalization. The primer sequences of the genes are shown in Table 3.

### 2.13. Statistical Analysis

The data in this study are shown as mean ± standard deviation (SD). Each experiment was repeated at least 3 times. The data were assumed to follow a normal distribution and analyzed and plotted using GraphPad Prism 8.0 (GraphPad Prism Software, Inc., San Diego, CA, USA). When *p* < 0.05, the difference was considered statistically significant, and when *p* < 0.001, the difference was considered statistically highly significant.

## 3. Results

### 3.1. ORY-1001 Enhances the Retinal Electrophysiological Function to Light Stimulation in rd10+ORY Mice

**Figure 1:**
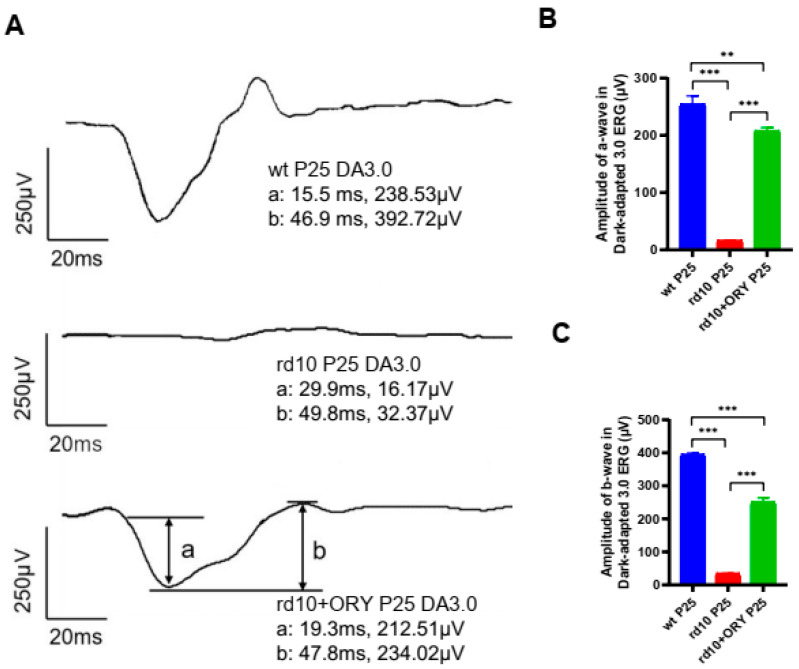
ORY-1001 improves retinal light responses in rd10 mice at P25. (**A**) Representative electroretinogram (ERG) trace in response to a light flash with scotopic 3.0 cd∙s/m^2^ stimulation under dark adaptation from a mouse (wt, rd10, and rd10+ORY). (**B**) Peak amplitudes of the scotopic a-waves. (**C**) Peak amplitudes of the scotopic b-waves. Data are shown as mean ± SEM (*n* = 6). ** *p* < 0.01, *** *p* < 0.001. P: postnatal day; wt, wild-type; rd10, rd10; rd10+ORY, rd10+ORY-1001.

To examine the effect of ORY-1001 on retinal physiology in rd10+ORY mice, ERG recordings were performed on each group of mice after 12 h of dark adaptation at P25 (Figure 1A). Using the response of neurons in the retina of wt mice to flash stimuli (a-wave appearance time was 15.5 ms, and a-wave amplitude was 238.53 μV; b-wave appearance time was 46.9 ms, and b-wave amplitude was 392.72 μV) for comparison, the response of the retinal neurons in the rd10 group decreased, which was related to the degeneration of retinal photoreceptors (a-wave appearance time was 29.9 ms, and a-wave amplitude was 16.17 μV; b-wave appearance time was 49.8 ms, and b-wave amplitude was 32.37 μV, Figure 1B,C). Compared with the rd10 group mice, the response in the rd10+ORY mice increased (a-wave appearance time was 19.3 ms, and a-wave amplitude was 212.51 μV; b-wave appearance time was 47.8 ms, and b-wave amplitude was 234.02 μV, Figure 1B,C). These results indicated that ORY-1001 can enhance the retinal response in rd10+ORY mice. However, we also noticed that there was still a difference between the rd10+ORY group and the wt group (Figure 1B,C), which meant that the effect of ORY-1001 was delaying but not blocking retinal degeneration.

### 3.2. ORY-1001 Improves Visual Behaviors in rd10+ORY Mice

**Figure 2:**
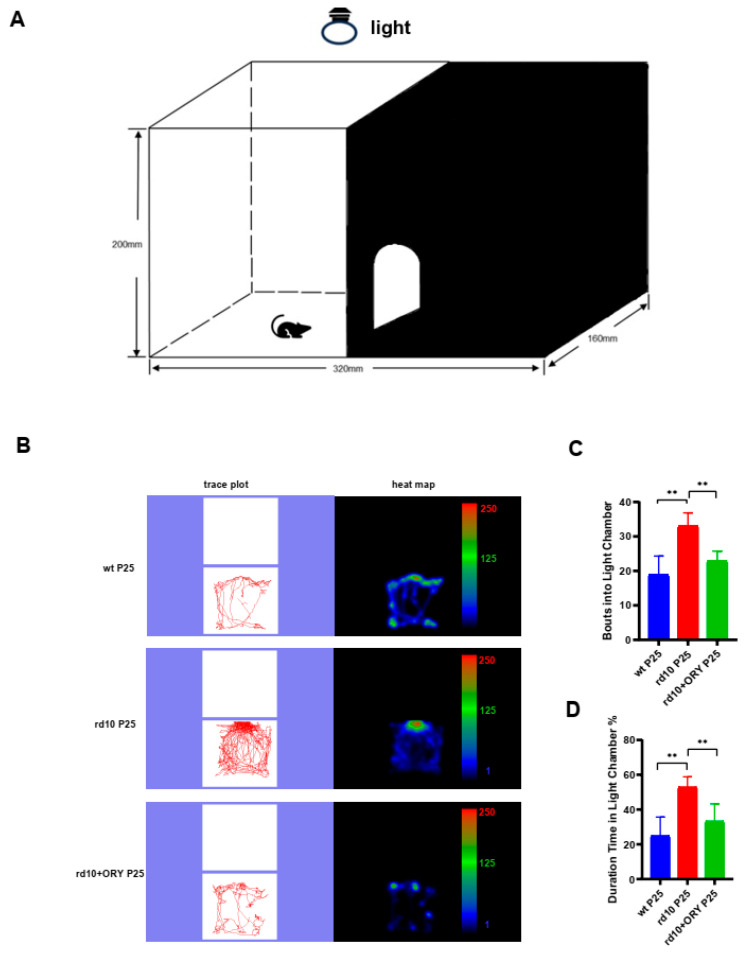
ORY-1001 improves visual behaviors in rd10 mice at P25. (**A**) Sketch map of a mouse test in the dark–light transition apparatus. (**B**) Representative trace plot of a mouse (wt, rd10, or rd10+ORY) in the light chamber. (**C**) Entries of a mouse (wt, rd10, or rd10+ORY) into dark chamber at P25. (**D**) Percentages of time in which a mouse (wt, rd10, or rd10+ORY) stayed in the light chamber (relative to the total duration of the test) at P25. Data are shown as mean ± SEM (*n* = 6). ** *p* < 0.01. P: postnatal day; wt, wild-type; rd10, rd10; rd10+ORY, rd10+ORY-1001.

To investigate whether using ORY-1001 could improve the visual function of rd10 mice, we performed the dark–light transition experiment to evaluate the mice’s perception of light (Figure 2A). At P25, the wt mice with normal vision spent less time in the white box (24.8 ± 9.8%) and entered and exited the dark and light boxes less frequently (18.8 ± 5). The rd10 group mice with degenerative photoreceptors spent more time in the white box (52.8 ± 5.5%, Figure 2B–D) and entered and exited the dark and light boxes more frequently (33 ± 3.5, Figure 2B–D). Compared with the rd10 group, the rd10+ORY group spent less time in the white box (33.2 ± 9.2%, Figure 2B–D), with less frequent entries and exits (22.8 ± 2.7, Figure 2B–D). There was no statistically significant difference in the test data between the wt and rd10+ORY groups (Figure 2B–D). The visual behavior test suggested that ORY-1001 improved the visual function of rd10+ORY mice.

### 3.3. ORY-1001 Preserves Retinal Structures in rd10+ORY Mice

**Figure 3:**
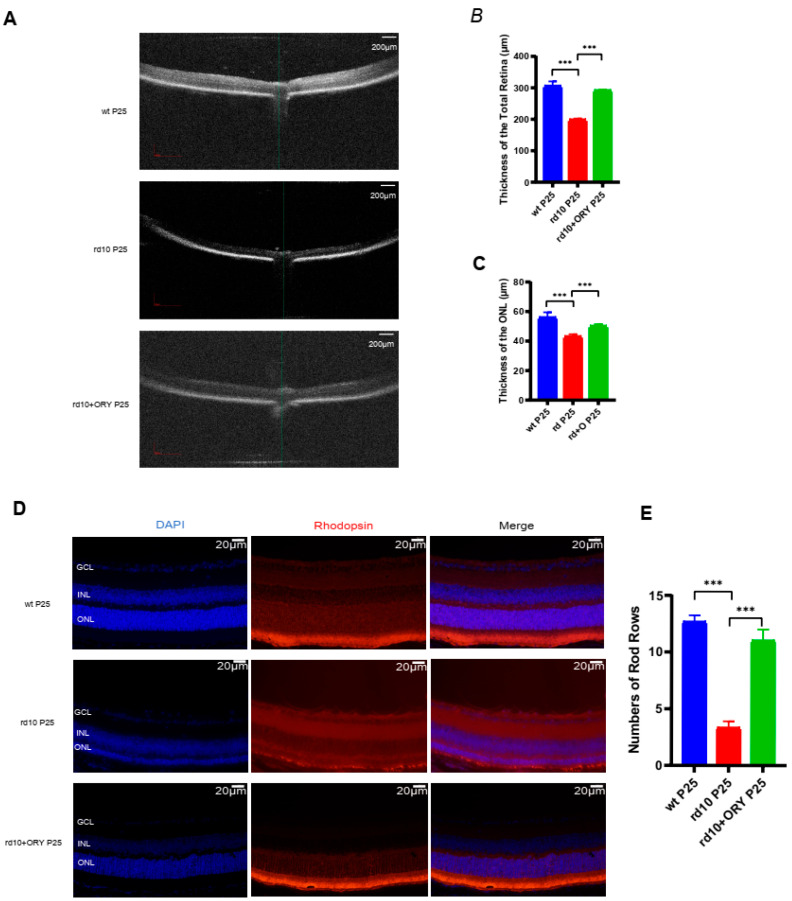
ORY-1001 preserves retinal structures in rd10 mice at P25. (**A**) Representative images of OCT from a mouse (wt, rd10, and rd10+ORY) in each group at P25. Scale bars: 200 μm. (**B**) Quantification of total retina thickness at a distance of 200 μm from the optic nerve head in each group at P25. (**C**) Quantification of ONL thickness at a distance of 200 μm from the optic nerve head in each group at P25. (**D**) Representative images of DAPI, Rhodopsin, and merge staining of retinal sections. Scale bars: 20 μm. (**E**) Number of rod rows in each group at P25. GCL: ganglion cell layer; INL: inner nuclear layer; ONL: outer nuclear layer. Data are shown as mean ± SEM (*n* = 6). *** *p* < 0.001. P: postnatal day; wt, wild-type; rd10, rd10; rd10+ORY, rd10+ORY-1001.

In order to investigate the protective effect of ORY-1001 on the retinal structure in rd10+ORY mice, OCT detection was performed on each group of mice at P25, and measurements were taken at a distance of 200 μ m from the optic nerve head (Figure 3A). Compared with the total retinal thickness (305.2 ± 13.9 μm) and outer nuclear layer thickness (55.7 ± 3.4 μm) of the wt group, the total retinal thickness (197.9 ± 4.2 μm) and outer nuclear layer thickness (42.0 ± 1.6 μm) of the rd10 group were thinner (Figure 3B,C). Compared with the rd10 group mice, the rd10+ORY group had greater total retinal thickness (290.9 ± 2.9 μm) and outer nuclear layer thickness (50.3 ± 1.4 μm) (Figure 3B,C). At P25, Rhodopsin immunofluorescence staining was performed on the retinal tissues of mice in each group (Figure 3D). There was a significant difference in the photoreceptor nuclear layer between the rd10 group and the wt group (Figure 3E), as well between the rd10+ORY and rd10 group (Figure 3E). These results indicated that ORY-1001 can protect the retinal structure in rd10+ORY mice.

### 3.4. ORY-1001 Increases the Expression Levels of H3K4me2 and CoREST in the Retinal Tissue of rd10+ORY Mice

**Figure 4:**
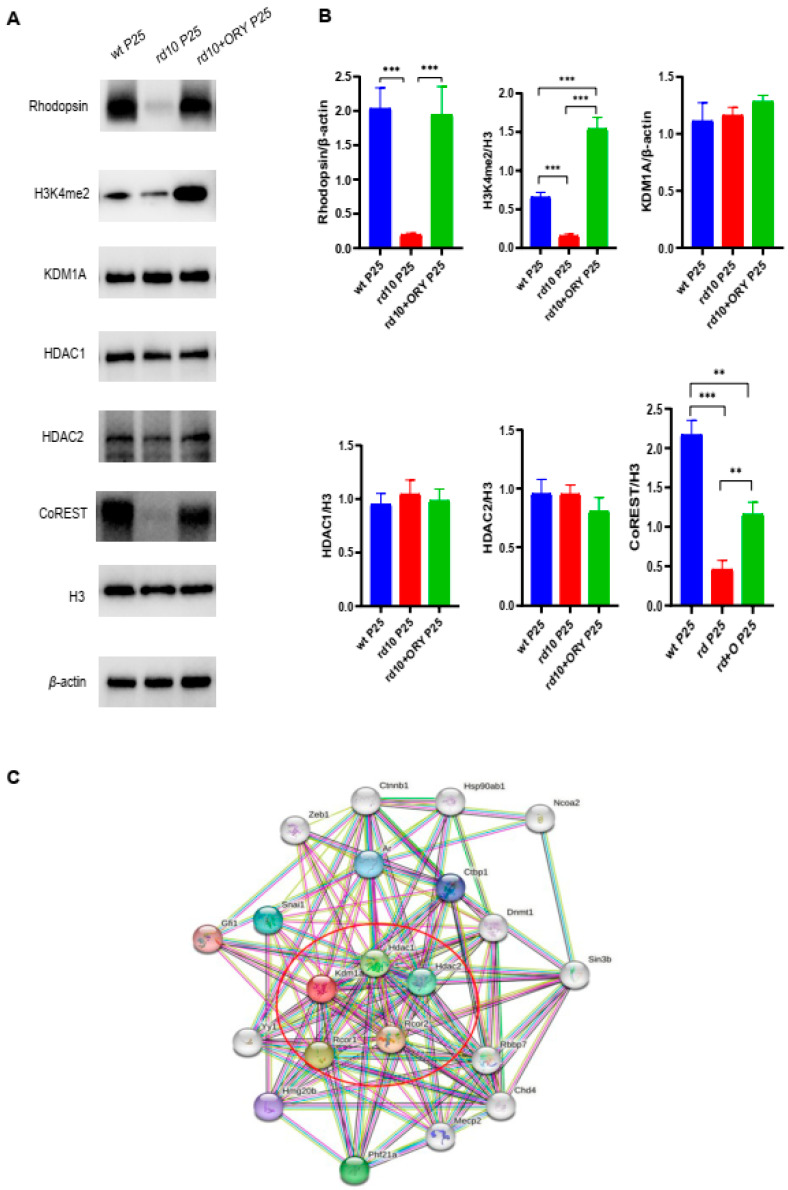
ORY-1001 increases the expression of H3K4me2 and CoREST in rd10 mice at P25. (**A**) Representative immunoblots of Rhodopsin, H3K4me2, KDM1A, HDAC1, HDAC2, and CoREST, with β-actin or H3 being used as a loading control (the original Western blot images are shown in Figure S1). (**B**) Statistical analysis of Rhodopsin, H3K4me2, KDM1A, HDAC1, HDAC2, and CoREST related to β-actin or H3. (**C**) Visualization analysis of protein directly interacting with KDM1A (from the STRING database, https://cn.string-db.org/cgi/network?taskId=bDTdcGOxNlQ3&sessionId=b7R4ERkM0KUh (accessed on 22 December 2025)). Data are shown as mean ± SEM (*n* = 6). ** *p* < 0.01, *** *p* < 0.001. P: postnatal day; wt, wild-type; rd10, rd10; rd10+ORY, rd10+ORY-1001.

To study the effect of ORY-1001 on the protein expression level of retinal tissues in rd10+ORY mice, Western blot was used to detect Rhodopsin, H3K4me2, KDM1A, HDAC1, HDAC2, and CoREST in the retinal tissues of mice in each group at P25 (Figure 4A). Compared with the wt group, the expression of Rhodopsin, H3K4me2, and CoREST in the rd10 group decreased, while there were no significant difference in the expression of KDM1A, HDAC,1 and HDAC2. Compared with the rd10 group, the expression of Rhodopsin, H3K4me2, and CoREST increased in the rd10+ORY group, but there were no significant differences in the expression of KDM1A, HDAC1, and HDAC2 (Figure 4B). These results suggested that ORY-1001 delayed the degeneration of retinal photoreceptors in rd10+ORY mice by increasing the expression levels of H3K4me2 and CoREST.

### 3.5. Conjoint Analysis of ATAC-Seq and RNA-Seq from the Retina Between the rd10 and rd10+ORY Group

**Figure 5:**
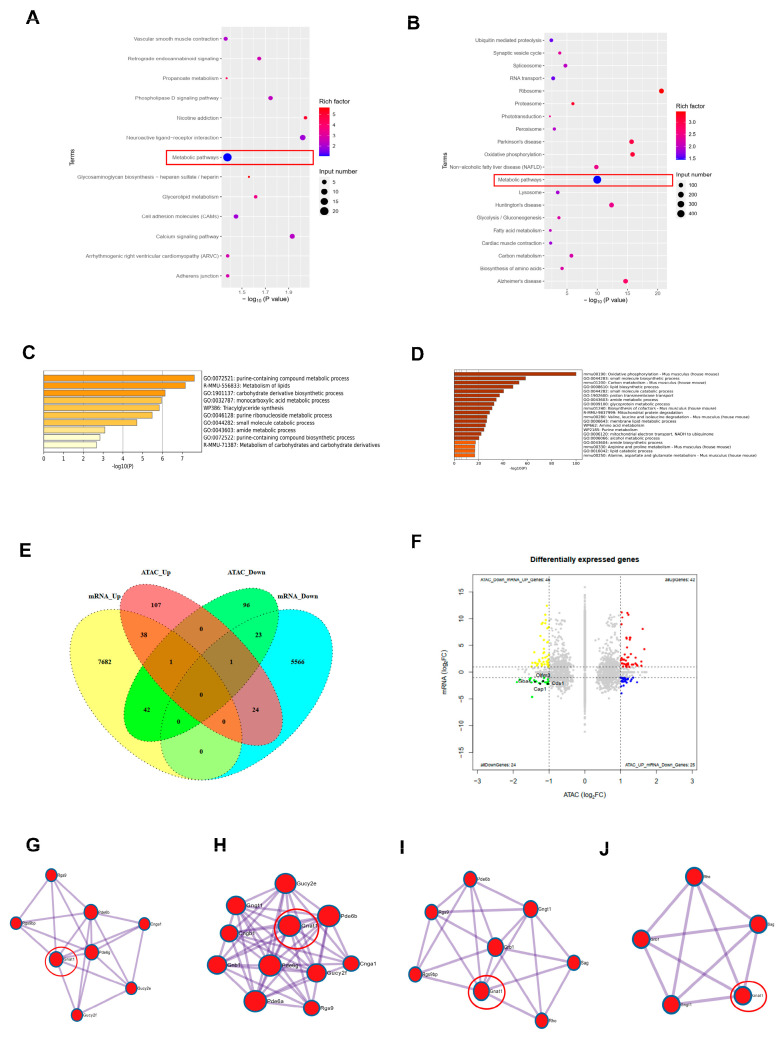
Visualization of conjoint analysis based on ATAC-seq and RNA-seq results between the rd and rd+O groups at P25. (**A**) Visualization of top items in KEGG analysis of ATAC-seq differential genes. (**B**) Visualization of top items in KEGG analysis of RNA-seq differential genes. (**C**) Visualization of ATAC-seq differential genes on metabolic pathway. (**D**) Visualization of RNA-seq differential genes on metabolic pathway. (**E**) Venn plot of conjoint analysis of ATAC-seq and RNA-seq differential genes. (**F**) Nine-grid plot of conjoint analysis of ATAC-seq and RNA-seq differential genes. (**G**) Visualization of RNA-seq visual perception pathway genes. (**H**) Visualization of RNA-seq visual phototransduction pathway genes. (**I**) Visualization of RNA-seq photoreceptor outer segment pathway genes. (**J**) Visualization of RNA-seq photoreceptor inner segment pathway genes. rd10, rd10; rd10+ORY, rd10+ORY-1001.

The previous results showed that ORY-1001 could delay the degeneration of retinal photoreceptors in rd10 mice, and increased the expression of H3K4me2 and CoREST. To understand the effect of ORY-1001 on retinal chromatin accessibility and gene expression, we performed ATAC-seq and RNA-seq analyze on retinal tissues from rd10 and rd10+ORY mice (Figure 5). A KEGG analysis was performed on the differentially expressed genes found in the ATAC-seq and RNA-seq results. Visualization showed that the differentially expressed genes were consistently enriched in metabolic pathways (Figure 5A,B), with 20 differentially expressed genes being enriched in metabolic pathways in the ATAC-seq results and 473 differentially expressed genes being enriched in metabolic pathways in the RNA-seq results. Visual analysis of these genes was carried out and shown (Figure 5C,D), which was by https://metascape.org/gp/index.html#/main/step1 accessed on 22 December 2025). It could be seen that ORY-1001 changed chromatin accessibility, which led to more gene expressions change, especially in metabolic pathways. The functions of these differentially expressed genes were involved in glucose, amino acid and lipid metabolism, and other metabolic pathways. The differentially expressed genes found in the ATAC-seq and RNA-seq results were conjoined and are displayed in Venn (Figure 5E) and nine-grid (Figure 5F) plots, in which the differentially down-expressed genes that were enriched in metabolic pathways are marked: Cap1, Cds1, Gbas, and Olmf3. These results indicated that ORY-1001 could reduce the expression of related genes in metabolic pathways. Our visual analysis revealed that the differentially down-expressed gene that was involved in retinal pathways in the RNA-seq results (Figure 5G–J) (https://metascape.org/gp/index.html#/main/step1 (accessed on 22 December 2025) was Gnat1.

### 3.6. Metabolomics Detection Results for Mrt and Mrd Confirming Metabolism Changes

**Figure 6:**
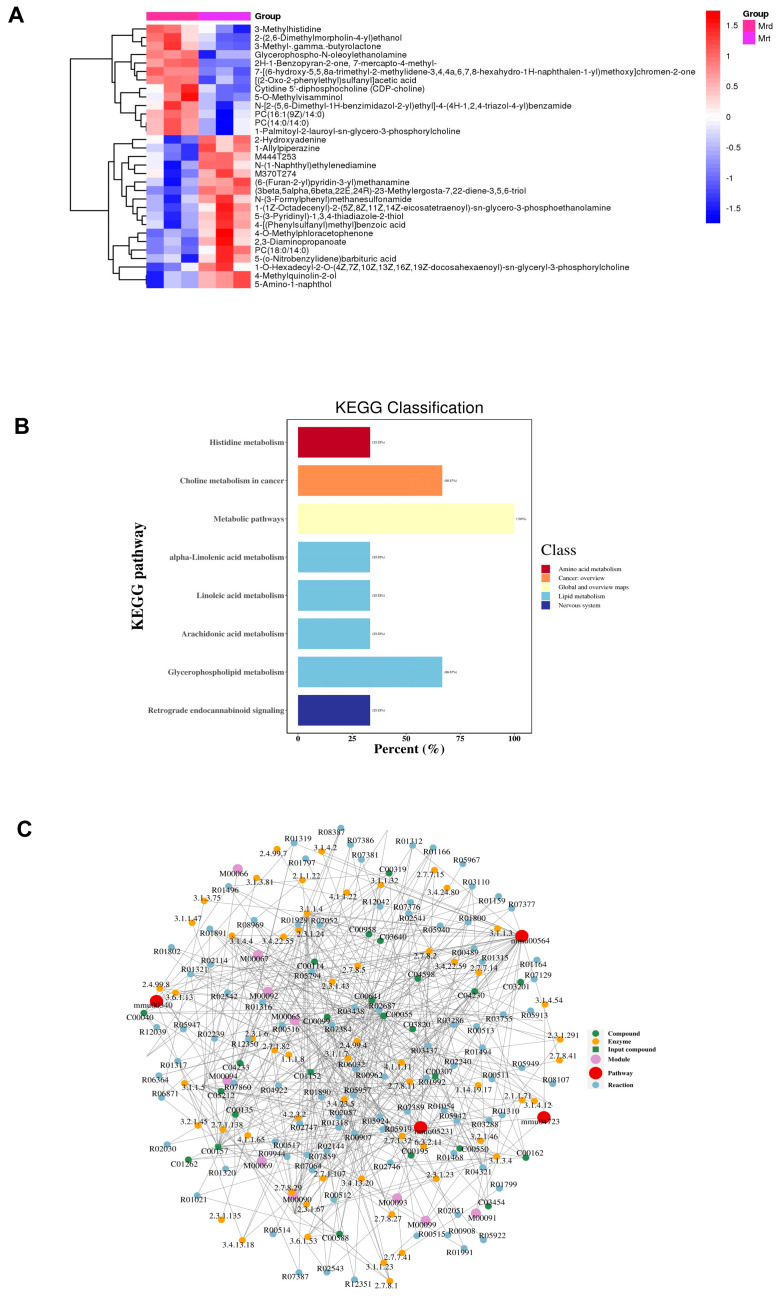
Differential metabolite analysis between rd10+ORY and rd10 groups. (**A**) Cluster heatmap of differential metabolites between rd10+ORY and rd10 groups. (**B**) KEGG analysis of differential metabolites between rd10+ORY and rd10 groups. (**C**) Analysis of differential metabolite regulatory networks between rd10+ORY and rd10 groups. Mrd10, rd10; Mrt, rd10+ORY-1001.

We analyzed the differential metabolites in the data for the rd10+ORY group (Mrt) and the rd10 group (Mrd), as shown in Figure 6. From the clustering heatmap analysis of differential metabolites between the two groups (Figure 6A), it can be seen that compared with the rd10 group (Mrd), the metabolite content in the rd10+ORY group (Mrt), including 3-methylhistidine, 2-(2,6-dimethylmorpholin-4-yl) ethanol, and 3-methyl-γ-butyrolactone, were obviously decreased. From the KEGG analysis of differential metabolites between the two groups (Figure 6B), it can be seen that differential metabolites are enriched in pathways such as metabolic pathways, amino acid metabolism, and lipid metabolism. From the analysis of the differential metabolite regulatory network between the two groups (Figure 6C), it can be seen that using ORY-1001 resulted in complex physiological and biochemical changes related to metabolism in retinal tissue, involving multiple enzymes, substrates, products, and pathways.

### 3.7. Validation of RNA-Seq Results by qRT-PCR

**Figure 7:**
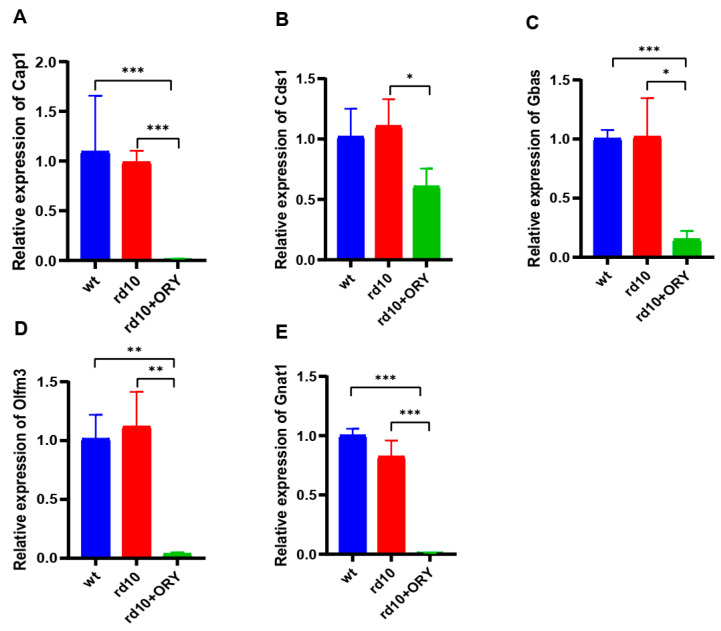
ORY-1001 reduces the expression of Cap1, Cds1, Gbas, Olmf3, and Gnat1. (**A**) Relative expression of Cap1. (**B**) Relative expression of Cds1. (**C**) Relative expression of Gbas. (**D**) Relative expression of Olmf3. (**E**) Relative expression of Gnat1. Data are shown as mean ± SEM (*n* = 12). * *p* < 0.05, ** *p* < 0.01, *** *p* < 0.001. wt, wild-type; rd10, rd10; rd10+ORY, rd10+ORY-1001.

qRT-PCR was used to confirm the relative expression of selective RNA from RNA-seq. The results verified the conclusions of the RNA-seq study (Figure 7). Compared with the rd10 group, the relative expression of Cap1 (Cyclase Associated Actin Cytoskeleton Regulatory Protein 1), Cds1 (CDP-Diacylglycerol Synthase 1), Gbas (Glioblastoma Amplified Sequence), Olmf3 (Olfactomedin 3), and Gnat1 (G Protein Subunit Alpha Transducin 1) were reduced due to the use of ORY-1001 in the rd10+ORY group (Figure 7A,E).

## 4. Discussion

RP is an inherited disease, which results in vision loss and eventually blindness due to the degeneration and death of rod and cone cells, which are the primary neurons in the visual transduction. The degeneration occurs in photoreceptors and is caused by genetic heterogeneity. Thus, the effects of current treatments for RP patients are quite limited, as existing gene therapy methods are only effective for specific patients [1,2]. It is therefore necessary to find non-specific potential therapeutic therapies that can delay the degeneration and death of photoreceptors [1,2].

Our study confirmed that the KDM1A inhibitor ORY-1001 could effectively delay the degeneration of retinal photoreceptors in rd10 mice based on the results of ERG electrophysiological detection, OCT detection, visual behavior detection, immunofluorescence staining and Western blot experiments. The detection and conjoint analysis of ATAC-seq and RNA-seq on retinal tissues of rd10 and rd10+ORY mice showed that chromatin accessibility was changed by using the KDM1A inhibitor ORY-1001, which in turn affected gene expression, especially of genes that are involved in metabolic pathways.

### 4.1. The KDM1A Inhibitor ORY-1001 Delays the Process of Retinal Photoreceptor Degeneration in rd10 Mice

Because existing evidence supported a reduction in H3K4me2 expression during retinal photoreceptor degeneration in rd10 mice [4], we used ORY-1001, a KDM1A inhibitor that can specifically inhibit H3K4me2 demethylation and increase H3K4me2 expression. As expected, our results proved that ORY-1001 could protect the structure and function of retinal photoreceptors in rd10 mice by increasing the expression of H3K4me2.

Our results proved that injecting ORY-1001 intraperitoneally at a dose of 0.075 mg/kg every second day during P14-P24 could effectively delay the degeneration and death of rod and cone cells in rd10 mice. The results of our ERG electrophysiological function test showed that ORY-1001 increased the response of rd10 mice to light stimulation under scotopic conditions. The dark–light transition test confirmed that ORY-1001 improved the visual behavior of rd10 mice. OCT detection and Rhodopsin immunofluorescence staining experiments both confirmed that ORY-1001 effectively protected the retinal structure in rd10 mice, especially the outer nuclear layer. In the results of Western blotting, the increased expression of Rhodopsin protein also explained the protective effect of ORY-1001 on retinal photoreceptors. We also discovered that the expression of H3K4me2 increased. All these data suggested that ORY-1001 can protect the structure and function of retinal photoreceptors in rd10 mice by increasing the expression of H3K4me2.

KDM1A, which is also known as LSD1, is a nuclear amine oxidase homolog, and that can demethylate mono- and/or demethylated H3K4 and/or H3K9, which results in regulating gene transcription [10,11]. The enzyme dynamically controls a series of biological processes by combining multiple protein complexes that regulate transcriptional activation and repression [10,11].

Histone methylation and demethylation modifications have played an important role in biological processes related to gene transcription regulation [10,11,12]. ORY-1001, as a highly selective, irreversible, and specific histone demethylase inhibitor on KDM1A, has previously inactivated KDM1A and inhibited H3K4me2 demethylation through irreversible binding with FAD cofactor [13]. Studies have confirmed that ORY-1001 can inhibit tumor cell proliferation and differentiation, induce tumor cell apoptosis, regulate the balance between stem cell self-renewal and differentiation by targeting the Notch pathway, and be used to treat acute myeloid leukemia and small cell lung cancer in clinical settings [5,6,7,8,9,12]. However, research focusing on the use of ORY-1001 in neurodegenerative diseases has not been reported. Our study was the first to confirm that ORY-1001 can effectively hinder the process of retinal photoreceptor degeneration in rd10 mice.

Our results demonstrated that there was no difference in the expression of KDM1A between the rd10+ORY and rd10 groups. KDM1A plays a role in the biological process by binding protein complexes. We searched in the string database and carried out visual analysis of KDM1A protein–protein interaction. The visualization showed that KDM1A, HDAC1, HDAC2, and CoREST are involved in direct protein–protein interactions with KDM1A. The results of Western blot showed that there were no statistical differences in the expression of KDM1A, HDAC1, and HDAC2 between the rd10+ORY and rd10 groups, but the expression of CoREST was increased in the rd10+ORY group. Studies have shown that in the process of epigenetic modification regulating histone methylation and demethylation, activating or inactivating protein complexes do not need to synthesize or degrade each member of the protein complex, but only regulate some key members [13]. Our results also confirmed this.

### 4.2. The KDM1A Inhibitor ORY-1001 Alters Chromatin Accessibility, Resulting in Metabolism Changes and Reduced Gene Expression in Metabolic Pathways

As an epigenetic modifier, ORY-1001 can specifically inhibit H3K4me2 demethylation and increase H3K4me2 expression. To explore the changes in chromatin accessibility and gene transcriptional expression caused by ORY-1001 contributing to the inhibiting of the degeneration of retinal photoreceptors in rd10 mice, we performed ATAC-seq and RNA-seq detection and carried out conjoint analysis on retinal tissues from the rd10 and rd10+ORY groups. The results showed that the differentially expressed genes were consistently enriched in metabolic pathways, while the gene expression were decreased. The ATAC-seq results showed that 20 differentially expressed genes were enriched in metabolic pathways, and the RNA-seq results showed that 473 differentially expressed genes were enriched in metabolic pathways. A visual analysis of gene functions showed that these differentially expressed genes are involved in glucose metabolism, amino acid metabolism, lipid metabolism, and other metabolic pathways. These results suggested that the inhibition of KDM1A by ORY-1001 changed the chromatin accessibility state significantly and thereby resulted in the down-expression of the genes in metabolic pathways that are involved in the metabolism of glucose, amino acids, lipids, and other substances. Visual analysis of differentially expressed genes on pathways related to the retina revealed that the effector gene was Gnat1. From our clustering heatmap analysis of differential metabolites, KEGG analysis of differential metabolites, and differential metabolite regulatory networks between the rd10+ORY and rd10 groups, it can be seen that the use of ORY-1001 resulted in complex physiological and biochemical changes related to metabolism in retinal tissue, involving multiple enzymes, substrates, products, and pathways. qRT-PCR also validated these results, which were drawn from the joint analysis of ATAC-seq and RNA-seq.

The differentially expressed genes Cap1, Cds1, Gbas, and Olmf3 on the metabolic pathways are involved in a range of physiological processes, such as intracellular cAMP production, lipid metabolism regulation, Ca^2+^ channel opening and closing, and retinal Progenitor cell development [14,15,16,17,18]. The Gnat1 gene encodes the α subunit of G protein transducin, which plays an important role in the process of retinal light transduction. It can affect the energy metabolism of the retina by changing its light regulation transduction [19,20,21]. Our study suggests that the epigenetic inhibitor ORY-1001 leads to genetic down-expression on the metabolic pathways and a reduction in retinal energy metabolism. The effector gene of retinal metabolism reduction was Gant1, which is also a key gene in the process of light transduction. The expression of Gnat1 was reduced, leading to the degeneration of retinal photoreceptors being impeded. Our experimental results are similar to those of previous studies, which also showed that metabolite therapy delays retinal degeneration [22].

In the future, we will need to detect the changes in retinal tissue metabolites, intervene in Gnat1, and investigate the relationship between Cap1, Cds1, Gbas, Olmf3, and Gnat1 for further research on the biochemistry mechanism of ORY-1001’s hinderance of retinal photoreceptor degeneration.

## 5. Conclusions

In summary, this study demonstrated that using the KDM1A inhibitor ORY-1001 can effectively delay the photoreceptor degeneration in the rd10 mouse model of RP, which is related to altering chromatin accessibility and gene expressions via epigenetic inhibitors. Therefore, regulating epigenetic modification may be a new strategy for the treatment of RP patients. However, as altering chromatin accessibility may significantly affect subsequent physiological processes, other relevant changes and impacts require further study.

## Figures and Tables

**Table 1:** List of reagents used in the experiment.

Name	Company	Catalog	Concentration
Rhodopsin	Abcam	ab98887	1:100
CoraLite594-conjugated Mouse IgG	proteintech	SA00014-5	1:100
DAPI	proteintech	28718-90-3	1:1000

**Table 2:** List of antibodies used in the experiment.

Name	Molecular Weight (kD)	Source	Company	Catalog	Concentration
H3K4me2	17	anti-rabbit	CST	9725S	1:1000
KDM1A	110	anti-rabbit	CST	2184S	1:1000
CoREST	66	anti-mouse	Santa Cruz	sc-376567	1:1000
HDAC1	62	anti-rabbit	CST	34589S	1:1000
HDAC2	60	anti-rabbit	CST	57156S	1:1000
Rhodopsin	38	anti-mouse	Abcam	ab98887	1:1000
H3	17	anti-rabbit	CST	4499S	1:1000
β-actin	43	anti-mouse	proteintech	66009-1-Ig	1:100,000
Goat anti-rabbit IgG-HRP		anti-rabbit	proteintech	SA00001-1	1:100,000
Goat anti-mouse IgG-HRP		anti-mouse	proteintech	SA00001-2	1:100,000

**Table 3:** Primer sequences of target genes.

Gene Name	Forward Primer Sequence	Reverse Primer Sequence
*Cap1*	CCAAACGCTCCTAAGCCAGA	CCACCTTAAGAGTTCCGCCT
*Cds1*	TGGACGCACGTCACTTTACT	GGCTCACACTCTGTCACGAA
*Gbas*	GAAGCCAGGTGTGGTAGCTT	TTCGCAGCCATTGGTACACT
*Olfm3*	GGGGACTGTTCTTTCAGCGA	CAAATGCATCGCCCATCAGG
*Gnat1*	TGTAGCCCTGTAGTCCCCTC	ACAGCATACCTTGACCAGCC
*β-actin*	GCAGGAGTACGATGAGTCCG	ACGCAGCTCAGTAACAGTCC

## Data Availability

ATAC-seq, RNA-seq, and metabolomics data will be provided during review.

## Supplementary Materials

The following supporting information can be downloaded at https://www.mdpi.com/article/10.3390/biology15020132/s1, Figure S1: Original Western blot image.

## Abbreviations

The following abbreviations are used in this manuscript:

RP	Retinitis pigmentosa
ff ERG	Full-field electroretinogram detection
OCT	Optical coherence tomography
qRT-PCR	Quantitative real-time polymerase chain reaction
KDM1A	Lysine-specific histone demethylase 1A
ORY-1001	Iadademstat dihydrochloride
ATAC-seq	Chromatin with high-throughput sequencing
LS-MS	Liquid Chromatography–Mass Spectrometry
KEGG	Kyoto Encyclopedia of Genes and Genomes
Cap1	Cyclase Associated Actin Cytoskeleton Regulatory Protein 1
Cds1	CDP-Diacylglycerol Synthase 1
Gbas	Glioblastoma Amplified Sequence
Olfm3	Olfactomedin 3
Gnat1	G Protein Subunit Alpha Transducin 1
