# Supplementary figures and images for: ORY-1001 Delays Retinal Photoreceptor Degeneration in rd10 Mice by Inhibiting H3K4me2 Demethylation
Source: Biology (Basel). 2026 Jan 13;15(2):132. doi: 10.3390/biology15020132 (PMC12837531; doi:10.3390/biology15020132)

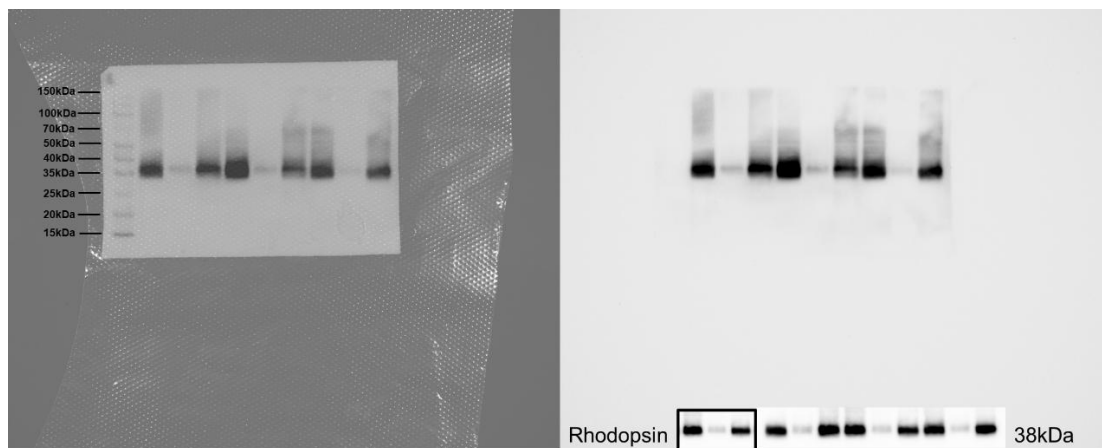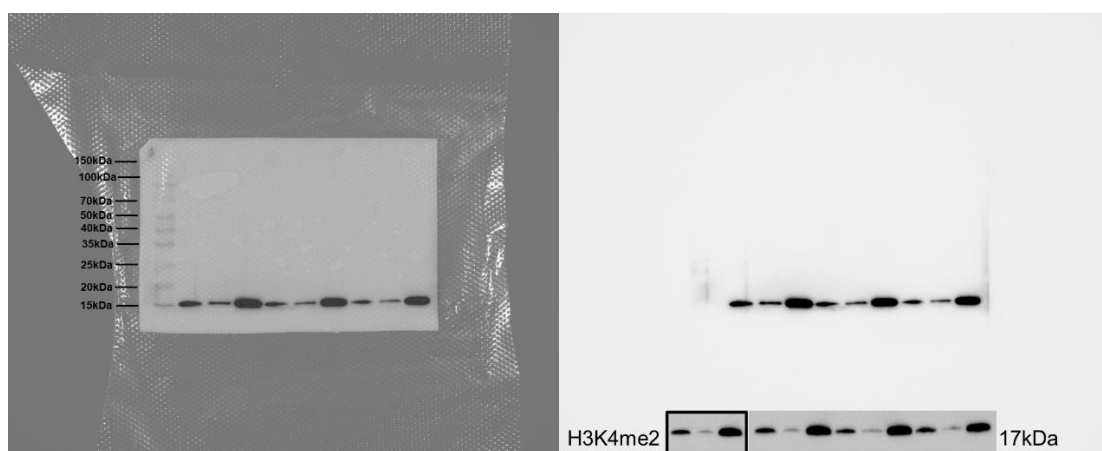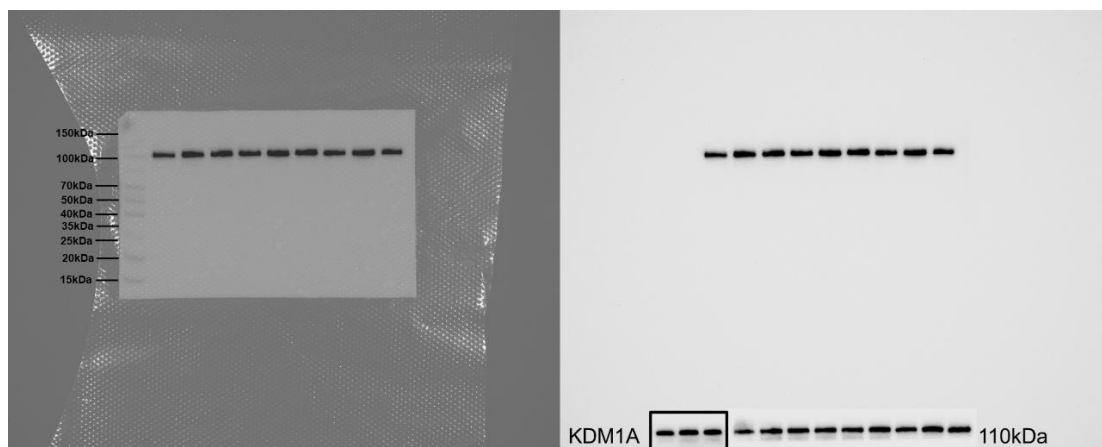

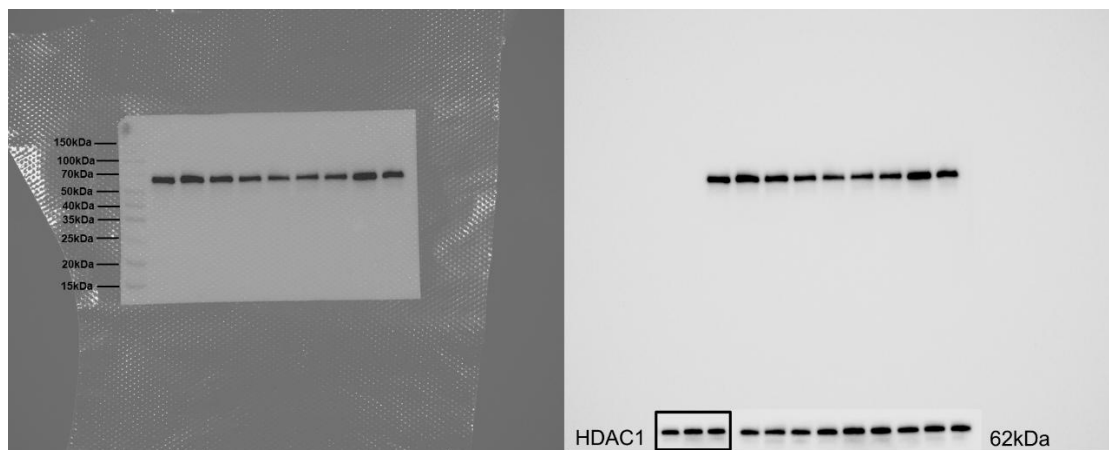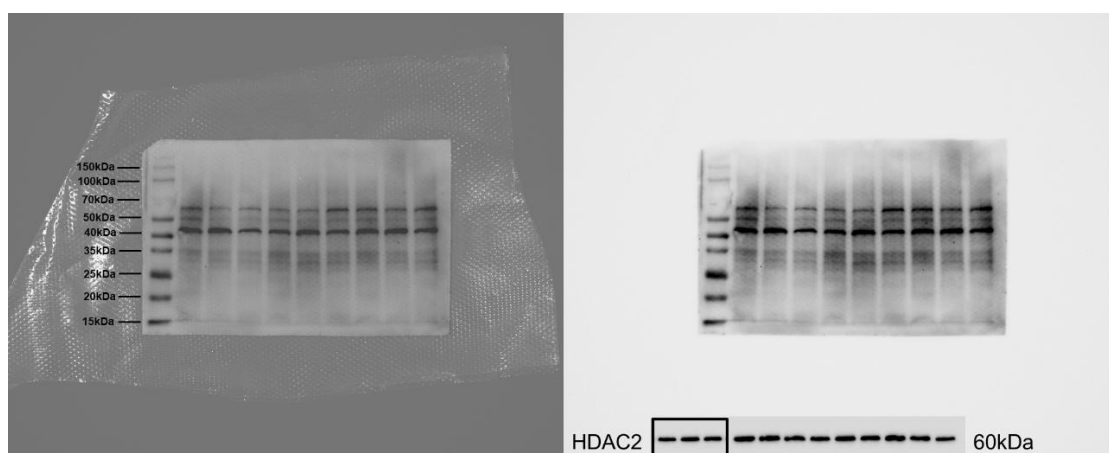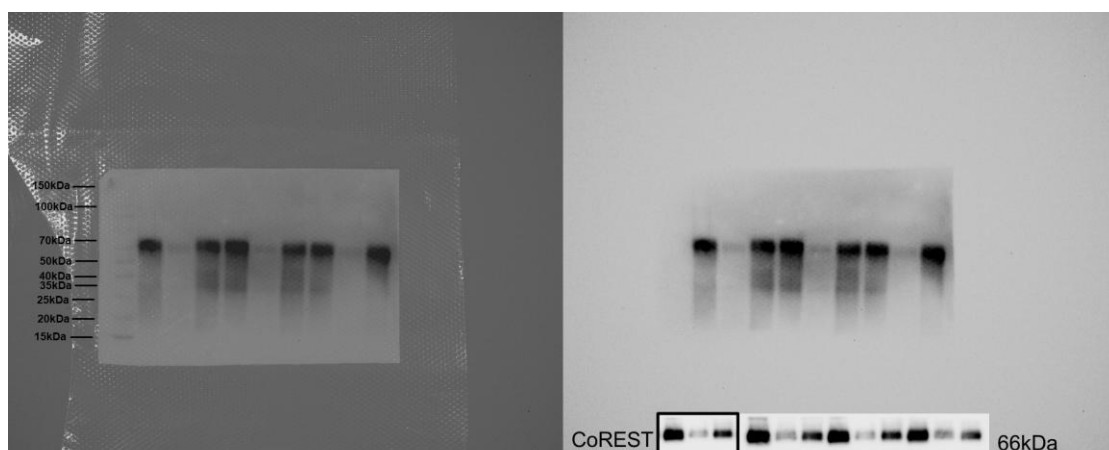

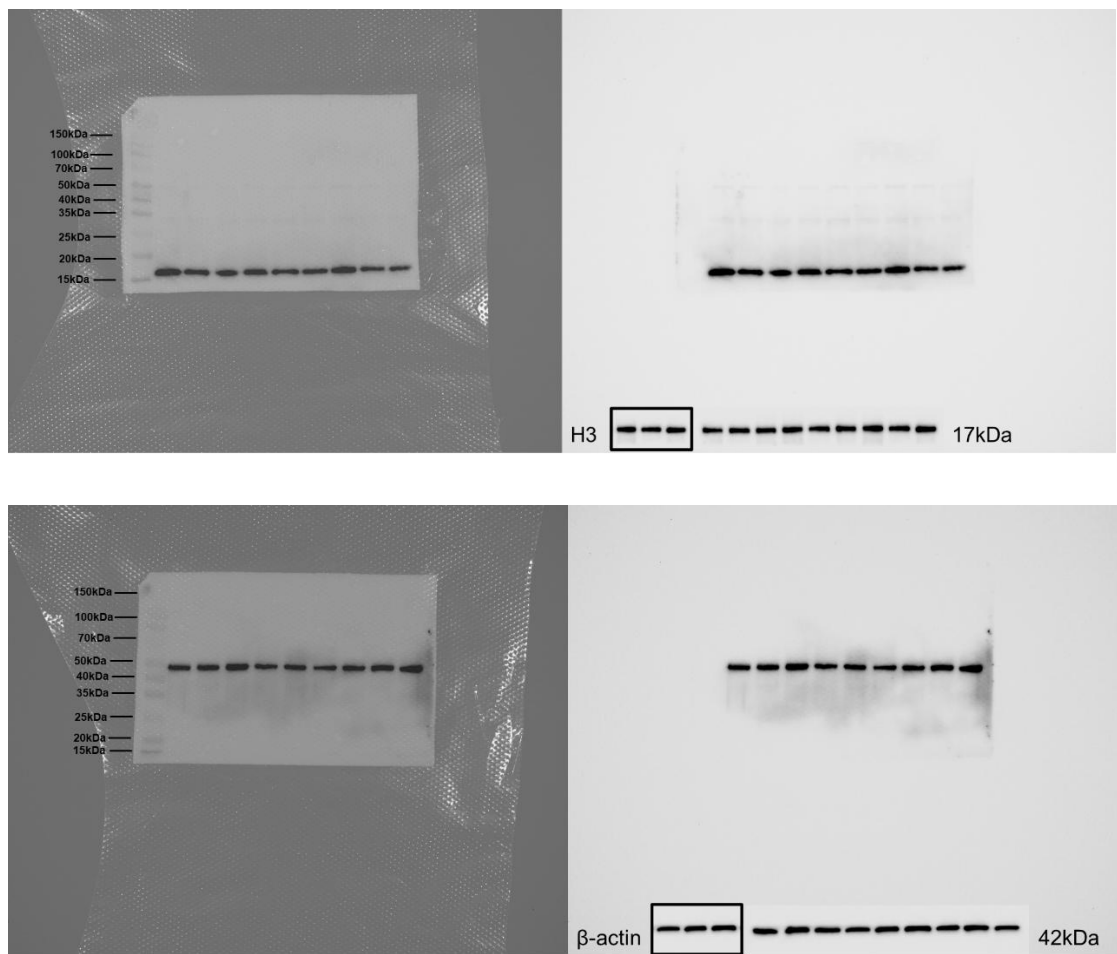

Figure S1: Original Western blot image.

Supplement: Supplementary file 1 [file biology-15-00132-s001.zip › biology-4021246-SI.pdf]
